# Sequence-specific bias correction for RNA-seq data using recurrent neural networks

**DOI:** 10.1186/s12864-016-3262-5

**Published:** 2017-01-25

**Authors:** Yao-zhong Zhang, Rui Yamaguchi, Seiya Imoto, Satoru Miyano

**Affiliations:** The Institute of Medical Science, The University of Tokyo, Shirokanedai 4-6-1, Minato-ku, Tokyo, 108-8639 Japan

**Keywords:** RNA-seq, Recurrent neural network, Sequence-specific bias, Gene expression analysis

## Abstract

**Background:**

The recent success of deep learning techniques in machine learning and artificial intelligence has stimulated a great deal of interest among bioinformaticians, who now wish to bring the power of deep learning to bare on a host of bioinformatical problems. Deep learning is ideally suited for biological problems that require automatic or hierarchical feature representation for biological data when prior knowledge is limited. In this work, we address the sequence-specific bias correction problem for RNA-seq data redusing Recurrent Neural Networks (RNNs) to model nucleotide sequences without pre-determining sequence structures. The sequence-specific bias of a read is then calculated based on the sequence probabilities estimated by RNNs, and used in the estimation of gene abundance.

**Result:**

We explore the application of two popular RNN recurrent units for this task and demonstrate that RNN-based approaches provide a flexible way to model nucleotide sequences without knowledge of predetermined sequence structures. Our experiments show that training a RNN-based nucleotide sequence model is efficient and RNN-based bias correction methods compare well with the-state-of-the-art sequence-specific bias correction method on the commonly used MAQC-III data set.

**Conclustions:**

RNNs provides an alternative and flexible way to calculate sequence-specific bias without explicitly pre-determining sequence structures.

**Electronic supplementary material:**

The online version of this article (doi:10.1186/s12864-016-3262-5) contains supplementary material, which is available to authorized users.

## Background

In recent years, deep learning techniques have been successfully applied to a number of challenging problems, including speech recognition, image processing, and machine translation. One major advantage of deep learning over traditional machine learning methods is the automatic extraction of a hierarchical feature representation for raw data and subsequent learning of a mapping to some desired output space. This is especially useful for those datasets for which manual feature extraction is difficult. In the bioinformatics community, more and more effort has been devoted to the application of deep learning methods to specific problems, such as gene expression [1, 2], alternative splicing [3, 4], gene regulation [5–8], and protein secondary structure prediction [9]. Progress of this kind has encouraged researchers to further explore other challenging bioinformatics problems in a deep learning way. In this work, we focus on the sequence-specific bias problem for RNA-seq data and proposed to solve it using RNNs.

RNA sequencing (RNA-seq) is a widely used whole-genome scale transcriptome profiling tool, which provides a high-throughput approach to measure gene and transcript expression for cell activities. For biological experiments using RNA-seq, many downstream analyses are heavily dependent on RNA-seq results. Therefore, the accuracy of expression estimation for RNA-seq data is important for downstream analysis.

Currently, most RNA-seq protocols require the preparation of a cDNA library, which coverts target RNA molecules to cDNA molecules for sequencing. In different stages of cDNA library preparation, such as RNA extraction and fragmentation, reverse transcription and amplification, different technical biases are incorporated. These biases inevitably effect the resulting expression estimation for RNA-seq data. Characterizing these technical biases and correcting their effect can go a long way to improve expression estimation accuracy. Here, we focus on one particular type of technical bias called sequence-specific bias. This bias, which happens when a read being selected for sequencing is affected by nucleotide sequences of primers, is introduced during reverse transcription due to differing binding efficiencies of random primers.

In the previous work, Hansen et al. [10] proposed to use a ratio of heptamer (7-mer) frequencies at the beginning and the middle position of a read as the sequence-specific bias weight. *Cufflinks* [11] uses a variable length Markov model to model sequence-specific bias and estimate the bias jointly with isoform expression estimation. Compared with Hansen’s method, *Cufflinks* takes the upstream nucleotides of a read’s aligned position into account and models position-specific bias cross transcript as well. Jones et al. [12] trained a Bayesian network with limited restrictions to determine the nucleotide sequence structure (nucleotide dependency topology in a sequence) that best distinguishes between biased and unbiased sequences. Read bias weights are then calculated based on the estimated sequence probabilities given the determined sequence structure.

In this paper, we propose to use Recurrent Neural Networks to estimate sequence-specific bias for RNA-seq reads. We are motivated to make use of the advantages of RNNs — namely that any arbitrary dependency of elements in a sequence can be automatically learned from data. Instead of explicitly determining sequence structure in advance, we use RNNs to characterize nucleotide sequences as a character-based language modelling task and calculate read bias weights according to RNN predicted sequence scores.

## Method

In this section, we describe the RNN-based sequence-specific bias correction pipeline for gene expression estimation (Additional file 1: Figure S1).

The pipeline can be divided into two major parts: (1) RNN-based bias estimation, and (2) gene expression quantification with bias reweighing. We describe each part in the following sections.

### RNN-based sequence-specific bias estimation for RNA-seq reads

In the process of cDNA library preparation, due to differing random primer binding efficiencies, a sequence-specific bias is introduced that results in the likelihood of a read being selected for sequencing is effected by the nucleotide sequence surrounding its read start-end.

To characterize sequence-specific bias, we follow a general approach introduced in previous work [10–12]. We sample a set of biased sequences and unbiased sequences, and train two sequence models for describing these sequences separately. The biased sequences are extracted surrounding the reference genomic coordinates where read start-ends are located. The unbiased sequences are extracted by randomly offsetting biased positions. We refer to biased sequences as foreground sequences, and unbiased sequences as background sequences. For a given read *r*, we use foreground and background models to calculate the sequence probability for the context nucleotide sequence of the read. The bias of the read is defined as the ratio of context genomic sequence probabilities as predicted by background and foreground models

$$\begin{array}{@{}rcl@{}} bias(r) = \frac{p_{f}(contextSeq(loci(r))}{p_{b}(contextSeq(loci(r))}, \end{array} $$

where *p*_*f*_ and *p*_*b*_ are foreground and background sequence probabilities, respectively. The function *contextSeq* returns the context genomic sequence surrounding the read start-end location *loci(r)* in reference genome.

The major difference among these types of sequence-specific bias correction methods is the model used to calculate nucleotide sequence probabilities. For example, Hansen et al. [10] counted hexamer (7-mer) positional statistics at the beginning and middle positions of a read as *p*_*f*_ and *p*_*b*_. In the 7-mer case, there are 16383 (4^7^−1) parameters for different positions so that the training data may not always be large enough to ensure accurate parameter estimation. *Cufflinks* [11] circumvents this problem by using a variable length Markov model, which only considered higher order dependencies for positions surrounding read start-end positions. Jones et al. [12] trained a Bayesian network to find principle structures of sequences that can be used to best distinguish between foreground and background sequences. Here, we propose to use RNNs for modeling sequence probabilities.

RNNs are artificial neural networks featured with directed-cycle connections between hidden neuron units. In the recurrent loop connection, shown in Additional file 1: Figure S1(c), a hidden unit from a previous time-step is connected to a current hidden unit. The information carried in the previous hidden unit is deemed as the “memory” of any previously visited sequence elements. Formally, at each time-step of the RNN an observed input vector *s*_*t*_  and hidden state vector  are used as inputs to calculate the current hidden vector *h*_*t*_ through the following recursive operation:

$$h_{t} = tanh(W_{s,h}s_{t}+ W_{h,h}h_{t-1} + b) $$

Here,  and  are model parameters. The output *y*_*t*_ is calculated with the *softmax* function on hidden units:

$$y_{t} = softmax(W_{h,y}h_{t} + c) $$

Here, *c* and *b* are bias units for the output and hidden layer, respectively.

Although an RNN has the ability to model arbitrarily long dependencies in theory, it is not easy to train an RNN, because of the non-linear connection between hidden units. In the backward step of the training phase, the gradient update at the end of a sequence may not be able to be back-propagated to the beginning of the sequence for a parameter update. This is called “gradient vanish” [13]. A commonly used strategy to solve this problem involves incorporating a gating mechanism for recurrent hidden units in an RNN, which forces a linear connection between recurrent units. In our pipeline, we use two types of gating RNNs: Long Short-term Memory Model (LSTM) [14] and Gated Recurrent Unit (GRU) [15].

Formally, given a trained foreground or background RNN model, the sequence probability of *S*=*s*_1_ *s*_2_…*s*_*m*_ is calculated according to

$$p_{f/b}(S) = p(s_{1}s_{2}\ldots {s}_{m}) = p(s_{1})\prod_{t=2}^{m}p(s_{t}|s_{1}\ldots {s}_{t-1}), $$

where *m* is the sequence length. The conditional probability *p*(*s*_*t*_|*s*_1_…*s*_*t*−1_) is expanded in the RNN as follows:

$$p(s_{t}|s_{1}\ldots {s}_{t-1}) = p(s_{t}|h_{t}\!) = \frac{exp(W_{h,s_{t}}h_{t} + c_{s_{t}})}{ \sum_{s' \in \{A,T,G,C\}}exp(W_{h,s'}h_{t} + c_{s'})} $$

The sequence probability factorizes element-wise for each time-step in the sequence, and each point-wise probability is calculated based on hidden units *h*_*i*_ in the current time-step.

### Bias correction for gene expression

After estimating sequence-specific bias weight for all reads, we correct the gene expression levels in light of sequence-specific bias. In the pipeline, after reads are mapped to a reference genome, we count uniquely mapped reads for each position in annotated exon regions. In the non-bias correction case, we can derive raw gene expression levels based on the counted reads in the gene with normalization. In particular, we perform FPKM normalization [16] normalized with gene length and the number of totally mapped reads:

$$ExpressLevel(gene) = 10^{9} \times \frac{\sum_{i}num({reads}_{<start\ at\ loci\ i >})}{length(gene) * num(reads) }, $$

where *i* is the exon genomic coordinate of the gene. In the bias correction case, the read counts are re-weighted with according bias weight in each position:

$${\begin{aligned} & ExpressLevel(gene) \\ &= 10^{9} \times \frac{\sum_{i}num({reads}_{<start\ at\ loci\ i>})/bias(contextSeq(i))}{length(gene) * num(reads) } \end{aligned}} $$

## Experiments and results

We evaluated the RNN-based sequence specific bias correction method using the MAQC-III dataset (GSE47774) [17]. In the dataset, each sample contains multiple expression level measurements from different technology platforms. We used the RNA-seq and qPCR data from *Universal Human Reference RNA* (UHRR) sample A and *Human Brain Reference* (HBRR) sample B for evaluation.

According to the pipeline, as shown in Additional file 1: Figure S1, we first aligned RNA-seq reads to the reference human genome (hg19/GRCg37) using “STAR” [18]. Over 87% of the approximately 100 million 100bp paired-end reads were uniquely mapped for samples A and B. Based on the alignment results, we counted the uniquely mapped reads at each position for all exonic regions and sampled sets of foreground and background sequences for our training RNN models using the sequence sampling function in the seqBias (1.20.0) package. It will be noted that we used the seqBias function to ensure that subsequent comparisons of our method to alternatives are fair. However, we can use our own sampling method in our implementation; see Additional file 1: Figure S1. In total, we sampled 100 k sequences for each of the training foreground and background models. These sequences are also used for training the Bayesian network based sequence-specific bias correction method seqBias. The sequence length is 21bp by default, which covers the 10 nucleotides before and after the read start-end positions in the reference genome. We filtered out any sequences near exon boundaries that are shorter than 21bp. We trained the foreground and background models in parallel, and estimated bias weights for the counted reads in exonic regions. The gene expression levels were estimated based on the re-weighted read counts using the estimated bias weights.

### RNN network scale

We trained two types of RNN models (LSTM and GRU) on the foreground and background sequences, respectively. The 100 k sequences are split into training/validation/test setsaccording to the proportions 90%:5%:5%. We used the character RNN language package^1^ to train our RNN models. The RNN parameters are tested for pre-defined parameter candidates and selected based on the minimum prediction error rate on validation data. As for the network scale, we use a “lightweight” RNN consisting of layer with the number of hidden nodes not more than 20 for each time-step. Based on trail and error, we found that increasing model complexity through the adding more layers and hidden nodes improves sequence modelling performance by lowering prediction error and perplexity ($Perplexity(S)=2^{-\frac {1}{N}\sum _{i=1}{N}log_{2}p(s_{i})}$, where *s*_*i*_ is a sequence in the test set *S* and *N* is the number of sequences in *S*. The lower perplexity is, the better model is). But for the sequence-specific bias correction task, the performance of correlations (correlation score between qPCR and predicted values) did not improves accordingly and significantly, while at the same time adding more computational cost. A simpler model is preferable on the grounds that it is more likely to be robust enough to capture the difference between foreground and background sequences and be less sensitive to the overfitting problem. Therefore, we choose “lightweight” RNN structures in our experiments. Table 1 shows training times of the GRU and LSTM models for different numbers of hidden units. For the sequence modelling aspect, the perplexities of RNN models on the test set decrease as more hidden number units are included.

**Table 1:** The number of model parameters and total training time for foreground models in 30 iterations

	GRU-10	GRU-20	LSTM-10	LSTM-20
Number of parameters	524	1644	684	2164
Training time	375.1 s	529.3 s	361.7 s	526.3 s
Perplexity of testing set	3.833	3.823	3.833	3.822

The training time is calculated on a 2.8 GHz CPU Macbook laptop where only the CPU is used

### Bias correction effect of RNN-based methods

To access the bias correction effect, we used the TaqMan RT-PCR data as the gold standard and evaluated the correlation between gene expression values (with/without bias correction) and corresponding non-zero RT-PCR values. We evaluated log-adjusted Pearson correlation, Spearman correlation, and *r* ^2^ values for the of goodness-of-fit for evaluation. We compared the performance of our method with the state-of-the-art seqBias method and the raw normalized counts.

As shown in Tables 2 and 3, both seqBias and RNN-based methods improve correlations between original predictions and RT-PCR values. For both sample A and B, GRU-based bias correction models (GRU-BC) yield results that are competitive with seqBias. In the evaluation with Pearson correlation and *r* ^2^ metrics, the GRU-based bias correction model with 10 hidden units performs slightly better, but this is not significant. The Pearson correlation between seqBias and GRU(4-10-4) is 0.9978 (log-scaled), and is 0.9989 between GRU(4-10-4) and LSTM(4-10-4), which indicates the three bias correction methods perform very similarly on the dataset.

**Table 2:** Correlation results on Sample A, replicate 1

Method	Pearson	Spearman	*r* ^2^
Raw normalized counts	0.8644	0.8708	0.7471
seqBias	0.8658	**0.8751**	0.7496
GRU(4-10-4)	**0.8674**	0.8749	**0.7523**
LSTM(4-10-4)	0.8661	0.8736	0.7460
GRU(4-20-4)	0.8661	0.8729	0.7500
LSTM(4-20-4)	0.8669	0.8744	0.7515

In total, 922 genes are evaluated. RNN(a-b-c) represents an RNN model with model structure defined according to *a, b*, and *c*. The values *a* and *c* represent the node number of the input and output layer, respectively. The value *b* is the number of hidden units. The boldface numbers indicate the best performance in all comparisons

**Table 3:** Correlation results on Sample B, replicate 1 for different sequence lengths. A total of 907 genes are evaluated

Sequences of 21bp length			
Method	Pearson	Spearman	*r* ^2^
Raw normalized counts	0.8758	0.8598	0.7670
seqBias	0.8764	0.8655	0.7681
GRU(4-10-4)	**0.8793**	**0.8664**	**0.7732**
LSTM(4-10-4)	0.8775	0.8624	0.7700
Sequences of 41bp length			
Raw normalized counts	0.8758	0.8598	0.7670
seqBias	0.8770	0.8665	0.7692
GRU(4-10-4)	0.8795	0.8663	0.7735
LSTM(4-10-4)	**0.8797**	**0.8689**	**0.7739**

The boldface numbers indicate the best performance in all comparisons

Differences can also be observed between the RNN-based approaches and seqBias. First, RNN-based bias correction methods are more conservative to change original predictions when compared with seqBias. The Euclidean distances between gene expression predictions before and after bias correction are 0.028 for GRU(4-10-4), 0.036 for LSTM(4-10-4), and 0.137 for seqBias. We think this is on account of the simple structure of RNNs that we have used. In Additional file 1: Figure S2, we observe that the RNN model bias corrected predictions are not too far away from the original predictions, when compared with seqBias. In the bottom-left region of the figure, we observe more differences for less abundant genes. Second, the proportion of increased and decreased changes in gene expression is also different. seqBias is right-skewed on the dataset with 867 decreased changes and 55 increased changes. In the histograms of log-fold changes in Additional file 1: Figure S3, the majority genes with decreased expression exhibit changes under zero, while for the RNN-based models, decreased changes and increased changes are approximately similar in a more balance proportion. Although which one is better can not be directly evaluated without knowledge of the true sequence-specific bias. That said, this result stands as an interesting difference between RNN-based bias correction methods and seqBias.

The sequence-specific bias of an RNA-seq read is examined by the context genomic sequence surrounding its read start-end. Usually, we make the length of the context window large enough to cover the positions that have a non-uniform positional nucleotide distribution. We extended the context window length from 21bp to 41bp on sample B and investigated whether longer context sequences effect bias correction results. As shown in Table 3, the performance of bias correction methods exhibit similar performance for both length scales. The LSTM-BC acquires additional improvements with longer sequences used as training samples. As LSTM has more parameters than GRU, extending the the context genomic sequence length incorporates more training data, which explains the improvement of LSTM-BC model. But the bias correction result does not improve significantly when compared with GRU-BC in Table 3.

## Discussion and conclusion

The biggest challenge in the sequence specific bias correction task is modelling the difference between foreground and background sequences. One simple approach is to describe the differences with a positional weight matrix that reflects the nucleotide distribution independently calculated at each position of the sequence. Although the sequence probability is easily calculated using a positional weight matrix, some complicate sequence patterns with differing foreground and background sequences might be ignored. On the other hand, directly counting whole sequences or enumerating long k-mers is liable to make the data too sparse to fit a model with a large number of parameters. To solve this trade-off, sequence structures are usually pre-determined heuristically or in data-driven way. RNNs make for an attractive alternative because information about past history is automatically encoded in hidden units so that no explicit structures need be determined in advance.

Of course, uncovering potential bias patterns in foreground sequences is no easy task. Training an RNN model on foreground sequences is more difficult than on background sequences, as the training and validation errors are prone to be higher on foreground sequences for the same model setting in our experiments. Simply increasing RNN model complexity runs the risk of overfitting, which, in this context, means that the model learns unexpected overfitted pattern for both background and foreground sequences. To mitigate this risk we used RNN models in a “shallow” way that use fewer layer and neurons for each recurrent hidden unit. What is more, shallow structures make training and testing an RNN model more efficient for the bias correction task.

In summary, we propose an alternative approach to current that employs RNNs to solve the sequence-specific bias correction problem for RNA-seq data. Our RNN-based model for bias weight estimation is lightweight and can be trained efficiently. The biggest advantage of this approach is that sequence structure is learned automatically during the training of RNNs. We conducted experiments on the MAQC-III dataset and achieved competitive results in comparison with the state-of-the-art Bayesian network method seqBias.

## Endnote

^1^ http://github.com/karpathy/char-rnn

## Additional file

Additional file 1 Supplement Figures. (PDF 577 kb)

## Abbreviations

GRU: Gated recurrent unit
LSTM: Long short term memory networks
MAQC/SEQC: MicroArray/sequencing quality control
RNNs: Recurrent neural networks
