# Supplementary material for: Sequence-specific bias correction for RNA-seq data using recurrent neural networks
Source: BMC Genomics. 2017 Jan 25;18(Suppl 1):1044. doi: 10.1186/s12864-016-3262-5 (PMC5310274; doi:10.1186/s12864-016-3262-5)
Supplement: Additional file 1 — Supplement Figures. (PDF 577 kb) [file 12864_2016_3262_MOESM1_ESM.pdf]

Supplement Figures

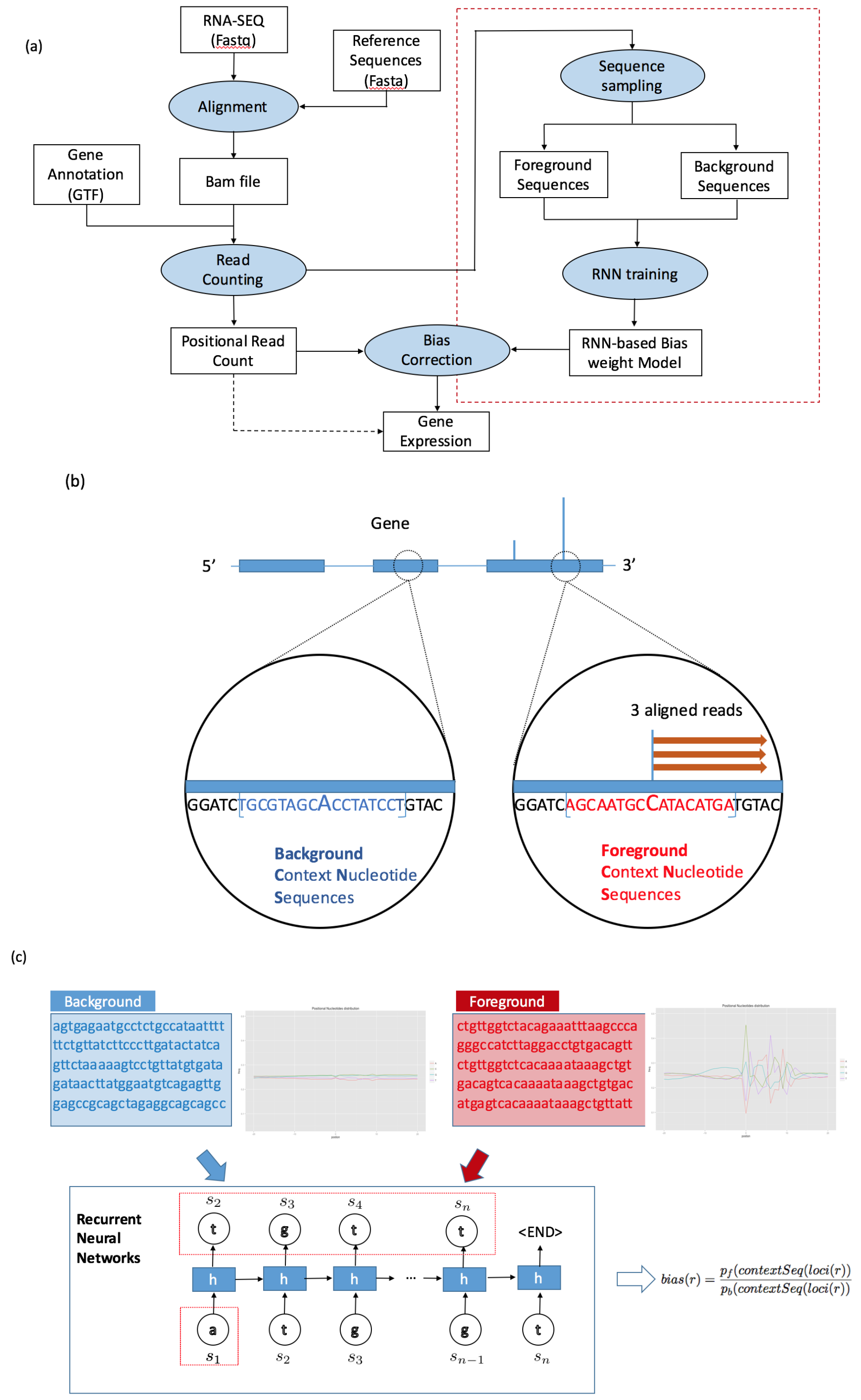

Figure 1: **RNN-based sequence-specific bias correction pipeline**. (a). The pipeline of RNN-based bias correction method for gene expression estimation. (b). An example of foreground and background sequences. Foreground sequences are extracted surrounding the read start-end positions and background sequences are extracted by randomly offsetting the selected read start-end positions. (c). Training RNN sequence models on foreground and background sequences. The probability of the sequence is calculated with the RNN prediction scores in the red rectangle except the initial position.

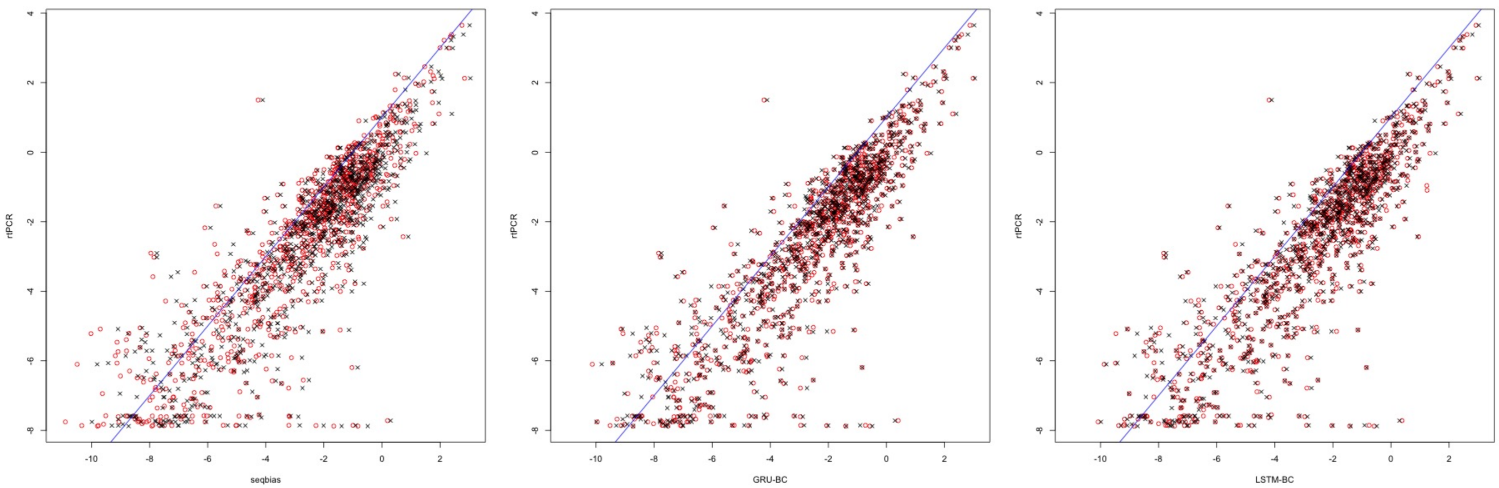

Figure 2: Scatter plot of gene abundant before and after bias correction on Sample A, replicate 1. The black crosses are predictions before bias correction and the red dots are the bias corrected predictions. The slope of the blue line is 1.

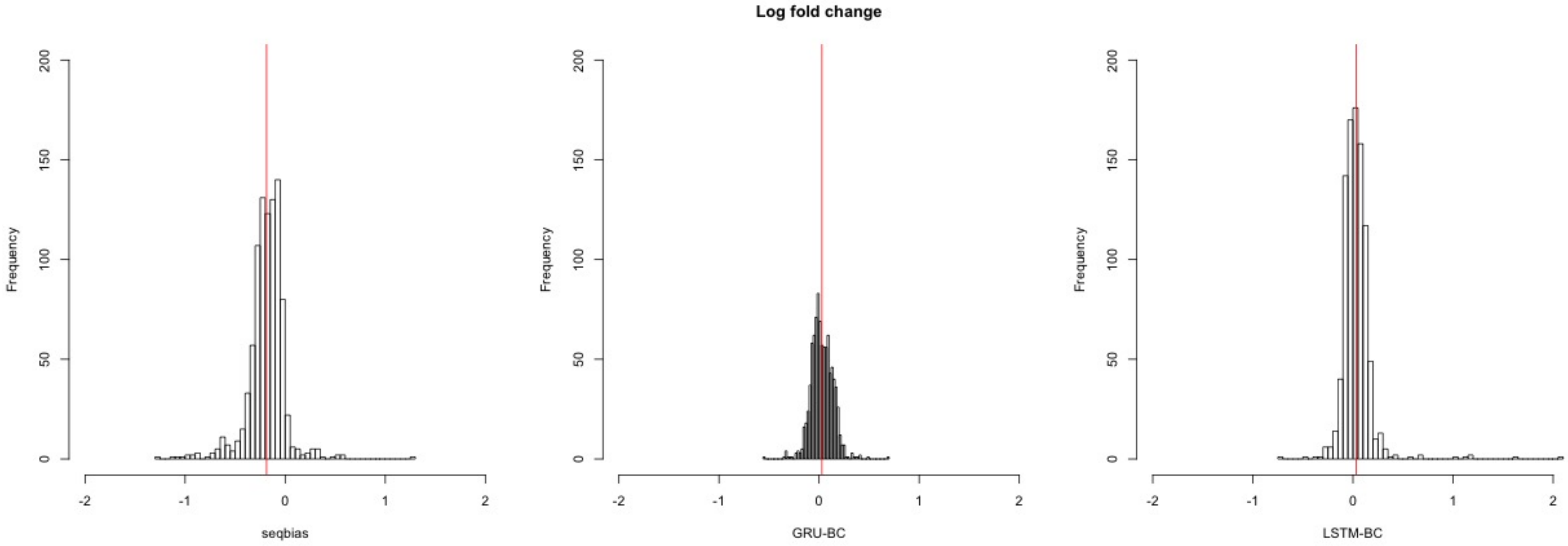

Figure 3: Log fold change in expression after bias correction.
